# E_L_FENN: A Generalized Platform for Modeling Ephaptic Coupling in Spiking Neuron Models

**DOI:** 10.3389/fninf.2019.00035

**Published:** 2019-05-31

**Authors:** Aaron R. Shifman, John E. Lewis

**Affiliations:** ^1^Department of Biology, University of Ottawa, Ottawa, ON, Canada; ^2^Center for Neural Dynamics, University of Ottawa, Ottawa, ON, Canada; ^3^uOttawa Brain and Mind Research Institute, Ottawa, ON, Canada

**Keywords:** electric field effects, LFP, modeling toolbox, bioelectric fields, phase locking

## Abstract

The transmembrane ionic currents that underlie changes in a cell's membrane potential give rise to electric fields in the extracellular space. In the context of brain activity, these electric fields form the basis for extracellularly recorded signals, such as multiunit activity, local field potentials and electroencephalograms. Understanding the underlying neuronal dynamics and localizing current sources using these signals is often challenging, and therefore effective computational modeling approaches are critical. Typically, the electric fields from neural activity are modeled in a *post-hoc* form, i.e., a traditional neuronal model is used to first generate the membrane currents, which in turn are then used to calculate the electric fields. When the conductivity of the extracellular space is high, the electric fields are weak, and therefore treating membrane currents and electric fields separately is justified. However, in brain regions of lower conductivity, extracellular fields can feed back and significantly influence the underlying transmembrane currents and dynamics of nearby neurons—this is often referred to as ephaptic coupling. The closed-loop nature of ephaptic coupling cannot be modeled using the *post-hoc* approaches implemented by existing software tools; instead, electric fields and neuronal dynamics must be solved simultaneously. To this end, we have developed a generalized modeling toolbox for studying ephaptic coupling in compartmental neuron models: E_L_FENN (E_L_ectric Field Effects in Neural Networks). In open loop conditions, we validate the separate components of E_L_FENN for modeling membrane dynamics and associated field potentials against standard approaches (NEURON and LFPy). Unlike standard approaches however, E_L_FENN enables the closed-loop condition to be modeled as well, in that the field potentials can feed back and influence membrane dynamics. As an example closed-loop case, we use E_L_FENN to study phase-locking of action potentials generated by a population of axons running parallel in a bundle. Being able to efficiently explore ephaptic coupling from a computational perspective using tools, such as E_L_FENN will allow us to better understand the physical basis of electric fields in the brain, as well as the conditions in which these fields may influence neuronal dynamics in general.

## Introduction

**Figure 1:**
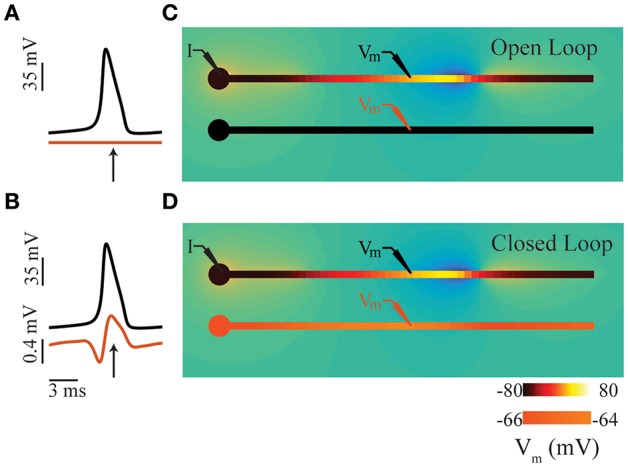
Intuition for ephaptic coupling. **(A)** Time series of membrane potentials in two adjacent neurons (comprising soma and axon compartments; see panel **(C)** in an open-loop condition. **(B)** Similar to panel **(A)**, except in closed-loop condition involving ephaptic coupling see panel **(D)**. The electric field generated by an action potential in the upper axon depolarizes the membrane potential of its neighbor. **(C)** Snap shots in time of open-loop condition representing a typical LFP simulation; time of snap shot is indicated by an arrow in panel **(A)**. **(D)** Similar to panel **(C)**, except in closed-loop condition involving ephaptic coupling; time of snap shot is indicated by an arrow in panel **(B)**. In all panels, a constant DC current is injected in the soma of the upper neuron (I) to produce propagating action potentials. In panels **(C,D)**, neuron color indicates membrane potential: in upper neuron, black denotes the resting potential (−80 mV), and yellow is the spike peak (+40 mV); the color scale for lower neuron is inflated by a factor of 16 for illustrative purposes. The transmembrane currents involved generate an electric field in the extracellular space (blue negative, orange positive); low extracellular conductivities were used for illustrative purposes, resulting in extracellular potentials ranging from −15 to 8mV.

Electrical signaling in nervous systems involves large transmembrane ionic currents that lead to the formation of electric fields. These electric fields can be recorded in a variety of ways at different spatiotemporal resolution, from single unit activity to local field potentials (LFPs) and electroencephalograms (EEG) (Buzsáki et al., 2012; Einevoll et al., 2013; Pesaran et al., 2018). In recent years, interest in the biophysical basis of these signals has increased, in part because of their importance in many brain-machine interface applications (Moran, 2010; Flint et al., 2013; Waldert, 2016). LFPs are generally characterized by the low-frequency (below ~100 Hz) components of extracellular signals thought to be dominated by synaptic processes (Buzsáki et al., 2012; Einevoll et al., 2013). To better understand these signals, many modeling approaches have been developed, often in the form of software packages, such as LFPy (Lindén et al., 2014), LFPsim (Parasuram et al., 2016), or VERTEX (Tomsett et al., 2014). Many of these packages are based on the NEURON simulation environment (Carnevale and Hines, 2006), but regardless of the underlying equation solver, the membrane potential and ionic currents are calculated first, and then subsequently (*post-hoc*), the extracellular potentials are calculated. Because the underlying neural dynamics are considered independent of the extracellular fields in this case, we refer to this *post-hoc* LFP computation as the open-loop condition (Figures 1A,C). In other words, the neural dynamics generate the LFP but the associated fields do not influence the neural dynamics. This scheme will be valid when the resulting fields are sufficiently weak, and the relevant timescales are short (Tveito et al., 2017).

However, when extracellular fields are sufficiently strong, they can affect membrane potentials, and in a sense, “feed back” and influence neuronal dynamics (Figures 1B,D); in such cases, the open-loop assumption is no longer valid. This closed-loop condition of the LFP is commonly referred to as ephaptic coupling (Arvanitaki, 1942; Jefferys, 1995; Holt and Koch, 1999; Anastassiou et al., 2010; Anastassiou and Koch, 2015). While the existence of ephaptic coupling is often acknowledged, it is not usually included in neuronal models. Indeed, this has been the subject of much debate (Weiss and Faber, 2010; Anastassiou and Koch, 2015). The common justification for omitting ephaptic coupling is that its influence is weak and insignificant. Recently however, weak ephaptic effects due to low-frequency oscillations (producing changes in membrane potential <0.5 mV) have been shown to strongly entrain neuronal activity (Fröhlich and McCormick, 2010; Anastassiou et al., 2011). And given that spiking probability can be dependent on the LFP phase (Gupta et al., 2016) and greatly influenced by noise transients (Kuhn et al., 2004; Destexhe and Contreras, 2006), the extracellular effects of local and population-level spiking activity could have a significant impact on neural computation.

In some contexts, weak electric field effects have been shown to play significant functional roles. For example, ephaptic coupling can modulate cardiac conduction velocity with respect to gap junctional connections, increasing conduction velocity when connectivity is weak and vice versa (Lin and Keener, 2010). Ephaptic coupling has also been implicated in the Mauthner cell system involved in the C-start escape response in fish, to enhance selectivity in response direction (Furukawa and Furshpan, 1963; Korn and Axelrad, 1980). Another interesting example has been described in some motor units, where ephaptic effects from muscle activity feeds back onto motor neuron axons, resulting in reverberating loops that enhance repetitive firing (Roth, 1994). Ephaptic coupling may also play a role in neuropathic pain through crosstalk between damaged nerves and adjacent fibers (Bridges et al., 2001; Cohen and Mao, 2014). And most recently, ephaptic coupling was shown to synchronize firing of cerebellar Purkinje cells (Han et al., 2018). In other systems, ephaptic coupling may be difficult to avoid. Weakly electric fish generate an oscillating field for electric sensing that permeates their entire body. The potential ephaptic effects which this relatively strong electric field has on neuronal processing is not known.

Although it is clear that ephaptic effects can be important in at least some conditions, the added complexity of extracellular influences on neurons in this closed-loop condition can lead to challenges from a modeling perspective; indeed, the tools required to compute ephaptic effects have not yet been packaged in any easy-to-use format. To this end, we have developed a general-purpose modeling toolbox to study ephaptic coupling that includes documentation and tutorials for various use cases.

## Existing Modeling Tools

From a neural dynamics perspective, there are a multitude of feature-rich compartmental model solvers: for example NEURON (Carnevale and Hines, 2006), GENESIS (Bower and Beeman, 1998), and BRIAN (Goodman and Brette, 2009). These solvers, as well as more specialized solvers (Tomsett et al., 2014), typically form the basis for *post-hoc* calculation of the LFP (or other bioelectric field measures). For example, LFPy (Lindén et al., 2014) is a popular tool that calculates LFPs [and now ECoG, EEG, and MEG signals (Hagen et al., 2018)] for neurons with geometries that can be as complex as the underlying NEURON environment can handle. In addition, BioNet is a recently developed tool which on top of enabling large simulations is also able to compute LFPs during NEURON simulations (Gratiy et al., 2018). None of these tools however are currently capable of easily integrating ephaptic coupling.

For ephaptic coupling (and extracellular stimulation in general), there are no software packages *per se*, but common methods involve the use of a resistive grid to describe the coupling of neurons in the extracellular space (Traub et al., 1985; Park et al., 2005), or in some cases the extracellular potentials are computed during the simulation and coupled directly to the neural dynamics (Holt and Koch, 1999; Stacey et al., 2015; Goldwyn and Rinzel, 2016). In the context of extracellular stimulation, finite element models implemented using COMSOL or equivalent tools are common (McIntyre and Grill, 2002; Elia and Lamberti, 2013; Joucla et al., 2014; Pelot et al., 2018). This set of techniques spans a broad spectrum of biological realism. On one side of this spectrum are one dimensional linear cable models (Goldwyn and Rinzel, 2016), which are the easiest computationally to evaluate. On the other side of the spectrum are finite element models (Joucla and Yvert, 2012), which even after standard approximations (Elia and Lamberti, 2013; Pelot et al., 2018) are computationally expensive, often making a detailed exploration of parameter spaces prohibitive. In addition, these model implementations are not always freely available and accessible to the general user; and in cases where models are published, they are often application specific. To this end, we have developed E_L_FENN (**E**_**L**_ectric **F**ield **E**ffects in **N**eural **N**etworks), a MATLAB (Mathworks.com) toolbox which provides an accessible interface to the Holt and Koch (1999) method of modeling ephaptic coupling. By using standard compartmental methods, notation and terminology (i.e., similar to that used in the NEURON environment), our goal is to make modeling ephaptic coupling accessible to the community at large.

## Modeling Ephaptic Coupling: Theory

In recent years, both ephaptic coupling (Holt and Koch, 1999; Stacey et al., 2015; Goldwyn and Rinzel, 2016) and extracellular stimulation (McIntyre and Grill, 2002; Joucla and Yvert, 2012) have been modeled computationally. Depending on the particular question, the methods vary from highly abstracted, but simple-to-evaluate linear cable models (Goldwyn and Rinzel, 2016) to highly detailed realistic neuronal morphologies implemented using computationally expensive finite element models (McIntyre and Grill, 2002) It is clear that neuron morphology plays a strong role in shaping the LFP (Lindén et al., 2010) and thus will influence ephaptic coupling dynamics. Here, we strike a balance between anatomical realism and computational expense and provide, based on previous work (Holt and Koch, 1999), a simple modeling interface to directly couple neuronal membrane potential dynamics and the LFP.

We begin with the Hodgkin-Huxley formalism for the transmembrane potential of a spatially-extended neuron (Hodgkin and Huxley, 1952; Dayan and Abbott, 2005). For a given neuronal compartment *i*, there is a membrane potential ($V_{m}^{i}$), a set of voltage-dependent ion channel conductances ($g_{ion}^{i}$, determined by their respective dynamical gating variables e.g., *m, h*, and *n*, not shown here) and equilibrium potentials (*E*_*ion*_); in addition, there is a current between any adjacent compartment *j*, through an intracellular resistance *R*^*i, j*^. The membrane dynamics for compartment *i* coupled to an adjacent compartment *j* are summarized in Equation (1). While it is true in general that a typical compartment would have at least two neighbors, we omit the second compartment here for simplicity:

(1) $$\begin{array}{l} C_{m}\frac{dV_{m}^{i}}{dt}+\underset{ion}{\sum }g_{ion}^{i}\left(V_{m}^{i}-E_{ion}\right)=\frac{1}{R^{i,\;j}}\left(V_{m}^{j}-V_{m}^{i}\right) \end{array}$$

For any given compartment, the transmembrane potential (*V*_*m*_), can be expressed as the difference between the intracellular potential (*V*_*in*_), and the extracellular potential (*V*_*out*_) (Equation 2):

(2) $$\begin{array}{l} V_{m}\equiv V_{in}-V_{out} \end{array}$$

If the extracellular potential *V*_*out*_ is 0 (more accurately, if the gradient in the extracellular potential is 0), then Equation 1 holds. In this situation, the axial current term is more appropriately described by the intracellular potential, leading to a modification of Equation (1):

(3) $$\begin{array}{l} C_{m}\frac{dV_{m}^{i}}{dt}+\underset{ion}{\sum }g_{ion}^{i}\left(V_{m}^{i}-E_{ion}\right)=\frac{1}{R^{i,\;j}}\left(V_{in}^{j}-V_{in}^{i}\right) \end{array}$$

Following previous methods (Holt and Koch, 1999; Goldwyn and Rinzel, 2016), we can rewrite Equations (2, 3) to incorporate non-zero extracellular potentials (Equation 4):

(4) $$\begin{array}{l} C_{m}\frac{dV_{m}^{i}}{dt}+\underset{ion}{\sum }g_{ion}^{i}\left(V_{m}^{i}-E_{ion}\right)=\frac{1}{R^{i,\;j}}\left(V_{m}^{j}-V_{m}^{i}\right)\\ +\frac{1}{R^{i,\;j}}\left(V_{out}^{j}-V_{out}^{i}\right) \end{array}$$

This extra term is referred to as the ephaptic current (Equation 5; (Holt and Koch, 1999)):

(5) $$\begin{array}{l} i_{eph}=\frac{1}{R^{i,\;j}}\left(V_{out}^{j}-V_{out}^{i}\right) \end{array}$$

Finally, we use a common formalism in both ephaptic and LFP literature to define *V*_*out*_ (Holt and Koch, 1999; Lindén et al., 2014). Under a quasi-electrostatic approximation, the extracellular potential will be the solution to the Poisson equation (Equation 6) where *V* is the potential over the extracellular domain, σ is the conductivity of the medium and *C*_*CSD*_ is the current source density arising from neural sources (Gratiy et al., 2017; Tveito et al., 2017).

(6) $$\begin{array}{l} \nabla \cdot \sigma \nabla V=-C_{CSD} \end{array}$$

**Figure 2:**
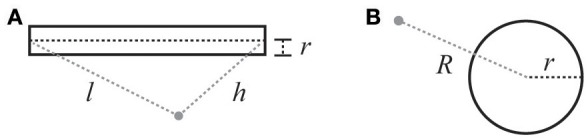
Illustration of variables for electric field calculations (see Equations 7 and 8). **(A)** Cylindrical current source: *r* is the radius of the cylinder, *l* is the distance from the beginning of the cylinder, h is the distance from the end. **(B)** Spherical current source: *R* is the distance from the center of the sphere, and *r* is the radius of the sphere.

While this equation can be solved using a finite element approach, as previously noted, this can be computationally expensive in practice. However, for certain boundary conditions these equations have explicit solutions. This is the case for standard compartmental neuronal geometries that use combinations of spherical and cylindrical compartments to model somata, axons, and dendrites. The solution for a cylindrical current source (Equation 7) comes from the line source approximation (Holt and Koch, 1999), and that for a spherical current source (Equation 8) comes from the point (spherical) source (see Figure 2 and legend for definitions).

(7) $$\begin{array}{lll} V_{out} & = & \frac{I_{m}r}{2\sigma }\log\left(\left|\frac{\sqrt{h^{2}+r^{2}}-h}{\sqrt{l^{2}+r^{2}}-l}\right|\right) \end{array}$$

(8) $$\begin{array}{lll} V_{out} & = & \frac{I_{m}r}{\sigma R} \end{array}$$

## Modeling Ephaptic Coupling: Implementation Using E_L_FENN

We have implemented the approach described in the previous section as a software toolbox called E_L_FENN (**E**_**L**_ectric **F**ield **E**ffects in **N**eural **N**etworks). E_L_FENN can be downloaded (see availability section) and installed from within MATLAB. E_L_FENN comprises two parts, a geometry toolchain and a solver toolchain.

To construct neuronal geometries, we use standard compartmental neuron modeling approaches, and for ease of use and comparison, standard terminology has been adopted where possible. As in the NEURON environment, model neurons are defined by a combination of spherical (somata) and cylindrical (axons and dendrites) geometries called sections, which are then discretized into nodes (where the equations are solved) called Segments.

To solve models with ephaptic coupling, we provide a wrapping solver which wraps commonly-used neural dynamics models with the extracellular dynamics. The resulting dynamics, as described above (Equations 3–7), form a system of differential algebraic equations (DAE). Extracellular potentials for each segment are given by Equations (7) or (8) (for cylindrical and spherical sections, respectively). Because the Poisson equation (Equation 6) is a linear PDE, linear superposition holds, i.e., the solution to the sum of several compartments (i.e., Segments) can be calculated as the sum of contributions from each individual compartment (Lindén et al., 2014; Parasuram et al., 2016). To calculate a single extracellular potential for each compartment, *V*_*out*_ is averaged over the boundary of the segment, which will be valid when the radius and length of the segment is small relative to the characteristic length of the field (at that point). Note that in some previous work, the cable equation is not discretized, leaving a partial DAE (PDAE), which is solved as a boundary value problem (Goldwyn and Rinzel, 2016).

The membrane dynamics depend on the extracellular potential (*V*_*out*_) (Equations 7 and 8), which in turn depend on the membrane current defined (Equation 4). Unlike traditional ODEs which when solved explicitly take the form of a function applied to the previous time step to compute the future time step, the circularity in this system requires the future time step to be solved self-consistently (similar to implicit methods). The DAE solver used here (MATLAB's ode15s) solves systems of the form:

(9) $$\begin{array}{l} Mẋ=\;f\left(x,\;t\right) \end{array}$$

where *M* is the identity matrix of equal dimension to that of the DAE with the exception that 0's on the diagonal specify the algebraic components. Given that all equations in the DAE are solved simultaneously, the time step is adjusted (shortened) until the numerical approximation of the DAE is satisfied to within tolerance, i.e., a self-consistent solution. The solver we have implemented here allows ephaptic dynamics to be included automatically without any extra work from the end user (see section Scope, Capabilities, and Limitations).

## Validating the Components of E_L_FENN

Given that E_L_FENN implements a combination of common algorithms, we can validate each component separately in open-loop conditions (i.e., no ephaptic coupling) against existing LFP and compartmental neuron model solvers. In the interest of brevity, we present only two comparisons here: action potential propagation using NEURON (to validate the membrane potential dynamics in open loop) and LFPy (to validate the LFP in open-loop); additional examples are included with E_L_FENN.

### Validating Neural Dynamics

**Figure 3:**
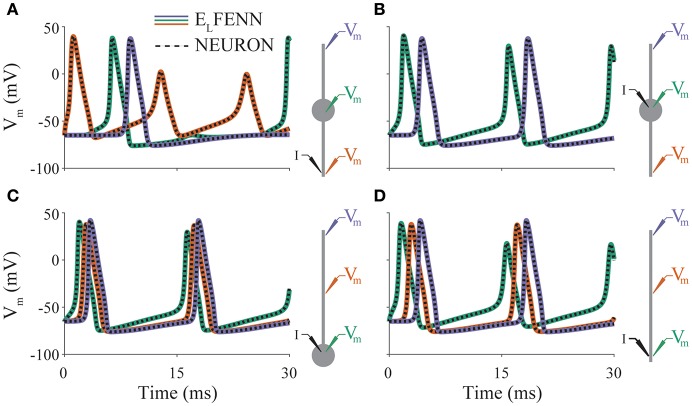
Validation of neural dynamics. Panels **(A–D)** show membrane potential over time for a different neuron morphology (indicated by schematics on right). Colored traces are E_L_FENN simulations at the recording locations denoted in schematic with appropriate color. Dashed traces are the corresponding NEURON solutions in each case. Current is injected at location denoted by I. Orange trace in panel **(B)** is occluded by purple trace; the solutions are identical due to symmetry.

In our first example (Figure 3), we consider action potential propagation in the classic Hodgkin-Huxley model included in NEURON (Carnevale and Hines, 2006). To validate the cable equation solver used in E_L_FENN, we implemented these dynamics using four basic neuronal morphologies with both E_L_FENN and NEURON (CVODE) solvers (in the E_L_FENN solver ephaptic coupling is turned off). Figures 3A–D shows a 30 ms simulation for each geometry, with membrane potential recordings at three representative locations (color-coded as green, orange, purple by location). A schematic of each neuron is presented on the right of each panel showing the recording locations along with the site of a constant current injection (I) used to initiate the repetitive action potentials. The solutions from E_L_FENN are color-code by location, and the solutions from NEURON are overlaid as a dashed black line in each case. The different solutions essentially overlap, with average RMS errors well below 0.5 mV, and the majority of error arising from slight discrepancies in spike timing (10–20 μs).

### Validating Generated LFPs

**Figure 4:**
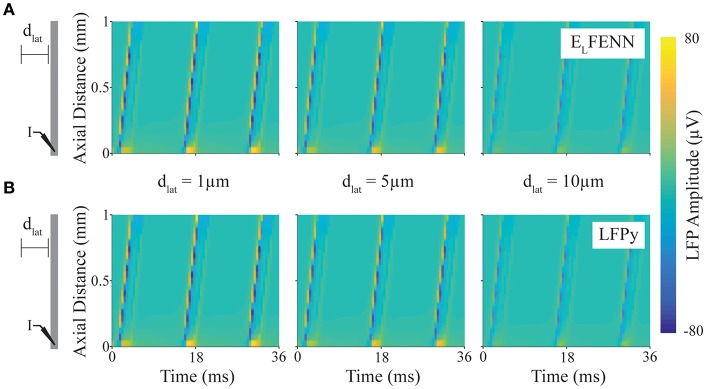
Validation of LFP calculation. Spatiotemporal LFPs from an axon firing repetitive action potentials calculated using LFPy or E_L_FENN (schematic shows DC current injection at “I,” and length of axon is scaled to plot). **(A)** LFP computed using E_L_FENN at lateral distances, d_lat_, of 1, 5, and 10 μm, respectively, from the axon. **(B)** LFP computed using LFPy at lateral distances, d_lat_, of 1, 5, and 10 μm, respectively, from the axon.

In our second example (Figure 4), we consider the same Hodgkin-Huxley model but limited to a single axon and calculate the LFP using LFPy (Lindén et al., 2014) to validate the LFP computed by E_L_FENN (in open loop). While only one geometry is presented here, all of the geometries considered in Figure 3 show similar results, and are included as additional comparisons with E_L_FENN software.

In Figure 4, a schematic of the setup shows a straight axon with DC current injected (I) at one end. LFPs are measured along a line parallel to the axon at lateral distances (d_lat_) of 1, 5, and 10 μm (note that we focus on the accuracy close to the axon boundary where *V*_*out*_ is determined). The upper (A) and lower plots (B) show the spatiotemporal LFP at these three locations, for E_L_FENN and LFPy, respectively. Contrary to many LFP plots which are in two spatial dimensions, only the vertical axis here represents space, with time represented on the horizontal axis. Wavelike behavior is apparent due to the periodicity of action potential firing caused by the DC current injection. Overall, the results of the two methods are quite similar with the RMS error of all results <1.7 μV (1.1%), and again the majority of the error arises from slight spike timing differences (~10 μs).

## Scope, Capabilities and Limitations

In this section we will describe the main components of the E_L_FENN toolbox. Further explanation and examples can be found in our documentation which can be accessed by running the MATLAB ‘doc’ command and clicking the E_L_FENN toolbox link once E_L_FENN has been installed.

### Using E_L_FENN: Geometry

The first step in the modeling process is to create a cell geometry. Using NEURON-like terminology, cells are composed of sections: cylindrical chunks for axons and dendrites, and spheres for somata. Cells in E_L_FENN are created section by section as seen in the following code snippet, where a spherical soma and a cylindrical axon are connected together to create a cell named ball_and_stick. Several additional functions for setting angles, orientations, and other morphological properties are explained in our documentation with examples.


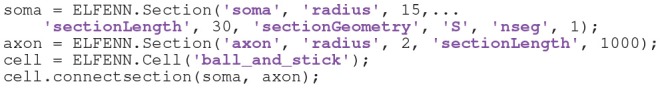


Alternatively, E_L_FENN is also able to import SWC files, of which a large database can be accessed from neuromorpho.org (Ascoli, 2006; Ascoli et al., 2007). The parser outputs an E_L_FENN compatible cell object (see section on realistic neuron example) which can then be tied to dynamics and simulated.

Once a cell, or multiple cells have been created they can be added to a network; for our purposes the word “network” refers to the whole system, i.e., whether the network is a single compartment or ten cells, the network label applies. In the following code, cell1 is added to the origin and a copy is added with a 100μm offset in the z-dimension.


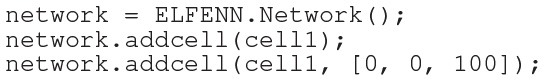


Much like cells, there are tools for setting orientation and position of networks that are described in our documentation.

### Using E_L_FENN: Dynamics

Action potential dynamics are typically modeled using two classes of models: continuous models, i.e., Hodgkin-Huxley-like where the dynamics are described by a set of differential equations and so-called hybrid models, such as the Izhikevich model (Izhikevich, 2003) or leaky integrate-and fire (LIF) models (Gerstner et al., 2014) which rely on discontinuities to generate spikes. E_L_FENN can only solve models of the continuous type. Any specific continuous model can be solved, as long as the dynamics can be set up as a function involving time, state, and parameters as inputs, and the right-hand side of the differential equations as a column vector of outputs. As an example, the source code for the Hodgkin-Huxley model is presented below.


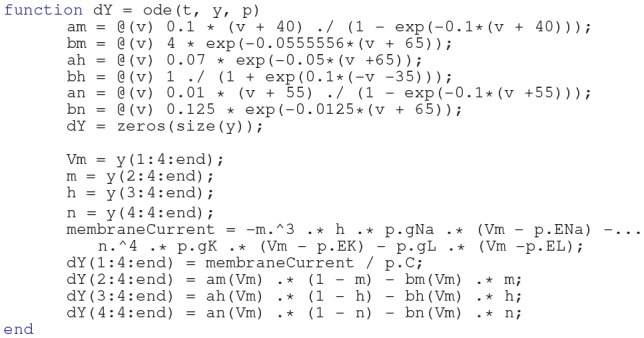


E_L_FENN includes three example dynamics with accompanying parameters: Hodgkin-Huxley, fast-spiking interneurons (Mancilla et al., 2007), and passive dynamics which require no custom code. However, any dynamics following the format of the above code snippet can be implemented.

### Using E_L_FENN: Synaptic Inputs and External Stimuli

E_L_FENN also includes a variety of features that allow synaptic interactions between neurons as well as external inputs (both synaptic and electrode-based, which can be applied to both the intracellular and extracellular space). For intracellular and extracellular stimuli, we include DC, and sinusoidal functions, however arbitrary stimulus functions can also be used, both autonomous (state dependent) and non-autonomous (time dependent). Extracellular stimuli can be either point current sources or parallel plate electric fields. Synaptic coupling between cells can involve gap junctions (electrical synapses) and/or chemical synapses. Lastly, E_L_FENN also provides functionality for external synaptic inputs with pre-set spike times (e.g., from a Poisson spike train).

### Using E_L_FENN: Limitations and Cost

Traditional compartmental models have an associated computational cost, and this will be true for any solver, including NEURON. Solving these models in the absence of ephaptic coupling will be primarily an order *n* operation [*O*(*n*)] since the axial current can be represented by a dot product of a sparse (almost) tridiagonal matrix (Hines, 1984). However, when incorporating ephaptic coupling, all currents will affect all compartments, and thus all pairwise interactions must be evaluated, i.e., an *O*(*n*^2^) operation. While any LFP solver (for example LFPy, LFPsim, or BioNet) will have the same *O*(*n*^2^) complexity they are not tied to the dynamical solution and therefore only need to be computed for successful time steps and can be down-sampled if required. These advantages are not available to E_L_FENN as the extracellular potentials are intrinsic to the dynamical system. Additionally, solving stiff DAEs (implicit methods), for which the Jacobian must be frequently estimated, and Newton iterations must be run to compute self-consistent values, can lead to high computational costs not typically seen when solving models without ephaptic coupling.

In terms of scalability, the aforementioned *O*(*n*^2^) complexity limits the size of the network (in terms of number of compartments) to whatever speed the user is willing to accept given their processor. Furthermore, because these algorithms in MATLAB are locked to a single processor, memory limitations are also a factor (e.g., with 8GB of RAM, 2,000–3,000 compartments might cause MATLAB to run out of memory). That said, much can still be learned about ephaptic coupling through the investigation of relatively small networks, for which the cost will be comparable to that of a standard model without ephaptic coupling solved using NEURON.

## Ephaptic Coupling in Realistic Geometries

**Figure 5:**
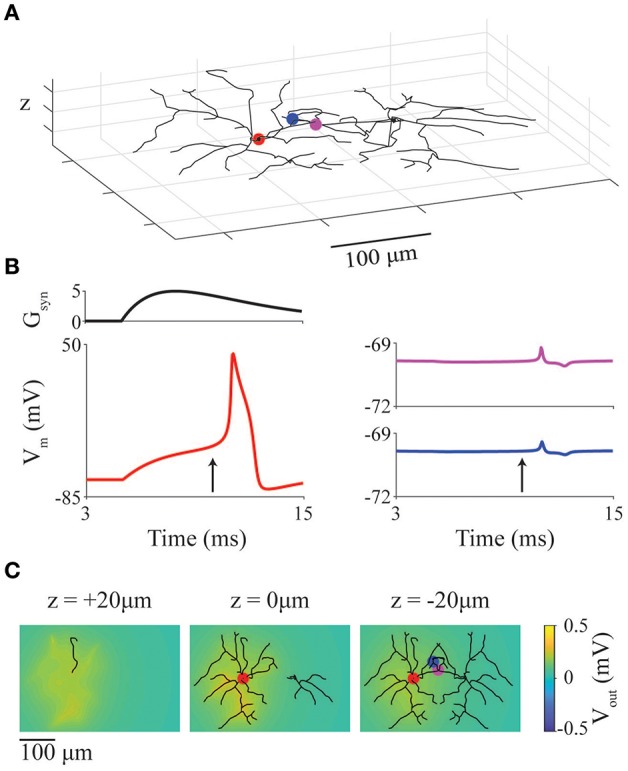
Ephaptic coupling between model neurons with realistic geometries. **(A)** Representation of two cortical interneuron models placed with somata 140 μm apart. Markers represent recording locations (red: active neuron, magenta and blue: inactive neuron). Red marker also represents location of a suprathreshold chemical synapse. **(B)** Synaptic conductance of synapse (G_syn_) at red point in panel **(A)** as well as membrane potential at red, blue, magenta points in panel **(A)**. **(C)** LFP at slices at *z* = 20, 0, −20 μm through the network at the time indicated by arrows in panel **(B)**.

As a simple example case, we present a model of two neurons with previously described stellate-like geometry (neuromorpho.org NMO_66468) (Ascoli, 2006; Ascoli et al., 2007; Morelli et al., 2017) and neural dynamics (Mancilla et al., 2007). The two cells are positioned in close-proximity, in an extracellular space with conductivity 0.01 S/m. The left (active) neuron has its soma at the origin ([0,0,0]), and the right neuron is mirror image and offset 140 μm to the right (Figure 5A). The soma of the left neuron receives a supra-threshold external synaptic input at 5 ms (location denoted by red marker). The red, purple and blue markers indicate compartments where membrane potential is recorded and displayed in Figure 5B.

In Figure 5B, the red traces are from the active (left) neuron (with synaptic conductance: upper trace) showing a full action potential; the blue and magenta traces are from the inactive (right) neuron and show the ephaptic coupling potentials (~500 μV deviation in membrane potential). To show the spatial heterogeneity of the extracellular potential (LFP), snapshots of the LFP (Figure 5C) are taken at 10 ms (vertical arrow in Figure 5B) and displayed as slices taken at z = 20, 0, and −20 μm.

The source code below highlights the simplicity of implementing this model using E_L_FENN:


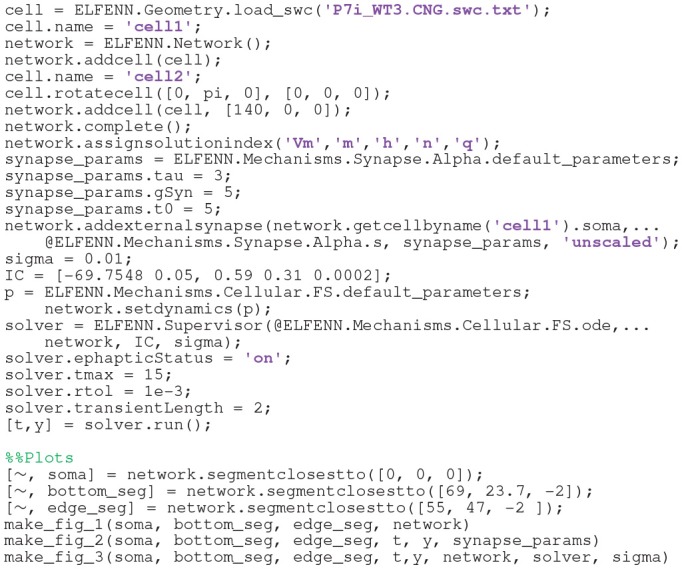


Note that the code for the plots do not relate to E_L_FENN specifically and as such are not displayed other than as function calls (see E_L_FENN documentation for full implementation).

## Ephaptic Coupling Between Adjacent Axons

We now present a simple use case for the novel closed-loop functionality of E_L_FENN: the ephaptic coupling among axons aligned in parallel (Ramón and Moore, 1978; Jefferys, 1995; Bokil et al., 2001; Goldwyn and Rinzel, 2016). In particular, we describe how the phase-locking effects of ephaptic coupling are influenced by extracellular conductivity in this situation. Although the range of conductivities we consider may exceed physiological values, we are interested in identifying the conditions for which ephaptic coupling could be functionally relevant. Conductivities can vary across brain region (Gabriel et al., 1996; Miceli et al., 2017) and may even be regulated, for example through the control of extracellular potassium by oligodendrocytes (Larson et al., 2018).

**Figure 6:**
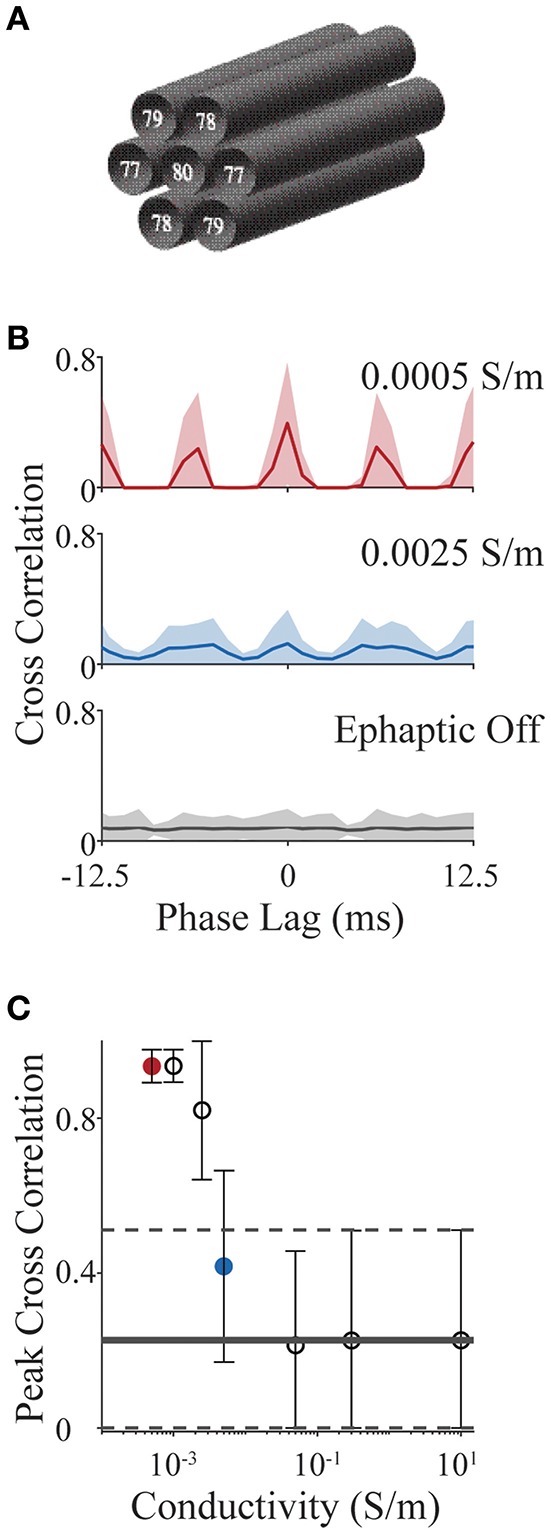
Action potential phase locking in an axon bundle through ephaptic coupling. **(A)** Schematic of 7 axons aligned in a bundle; base frequency for each is indicated in white text. All parameter values for all examples are provided with E_L_FENN software. **(B)** Cross correlation functions for strong ephaptic (red), weak ephaptic (blue) and no ephaptic conditions (gray). Data is presented as mean (solid line) ± standard deviation (shaded) over axon pair and initial conditions. **(C)** Effect of conductivity on phase locking. Peak cross correlation value (mean ± standard deviation) is plotted as a function of conductivity; red and blue symbols correspond to red and blue conditions in panel **(B)**; horizontal lines (solid and dashed) represent the “ephaptic off” case (mean and standard deviation, respectively).

We consider a simplified scenario involving an axon bundle, in which a central axon is surrounded by six others, hexagonally tiled (see Figure 6A). All axons have standard Hodgkin-Huxley dynamics (as in section Validating the Components of E_L_FENN; Figures 3, 4) and are set in an oscillatory firing regime, each with different baseline frequencies, by slightly different levels of DC current injection in the first segment (frequencies are annotated in Figure 6A). The initial condition for each axon is a random point on its own limit cycle to minimize effects of individual transients; the simulation time was 2 s. We use the spike train cross correlation function to characterize phase locking. In open-loop conditions (no ephaptic coupling), or when conductivity (σ) is very high, one would expect the spike trains to precess and thus cross-correlation functions would have a low uniform value on average across all phase lags. On the other hand, when firing is tightly coupled, there would be structure in the cross correlation e.g., dominant peaks at multiples of the locking period, potentially shifted by a phase lag. To measure the cross correlation, spikes in the last second of the simulation were binned into 1 ms windows (average oscillator period is 12.5 ms) and the cross correlation was computed and presented for ± 1 oscillator period. We vary conductivities (σ) from 10 S/m (well above typical CNS conductivities) to 0.05 S/m which is the lowest estimate of conductivity in the CNS across multiple studies (Gabriel et al., 1996; Miceli et al., 2017). We then extend the analysis to a value of 5 × 10^−4^ S/m as an upper bound on the effect size.

Figure 6B shows cross correlation functions for open-loop (ephaptic off; gray), intermediate conductivity (σ = 0.0025 S/m; blue) and low conductivity (σ = 5 × 10^−4^ S/m; red). Cross correlations are computed for each neuron pair (7 neurons yielding 21 unique pairs) over 15 sets of initial conditions; the data are presented as the mean correlation with shaded region showing standard deviation. For increasing ephaptic effect (decreasing conductivity), we see larger peaks at 0 and 6 ms (synchronous and anti-synchronous locking). To summarize these effects, the peak cross-correlation is presented in Figure 6C as mean and standard deviation across initial conditions and axon pair (all conductivities used the same sets of initial conditions). The red and blue symbols in Figure 6C show peak measures for the same data shown by the red and blue traces in Figure 6B, and horizontal line represents the mean cross-correlation value where ephaptic coupling is removed (standard deviation indicated by dashed lines); note that the peaks of the mean cross-correlations shown in Figure 6B will be in general much smaller than the peak correlations (Figure 6C) as they are averaged over different locking regimes for which peaks occur at different phase lags.

Overall, these results show that with increasing ephaptic strength (decreasing conductivity) there is an emergence of phase locking over the time scales tested (within 2 s). As in previous studies (Jefferys et al., 2012; Han et al., 2018), this demonstrates the potential for crosstalk between neurons that can lead to phase-locking. It is interesting to note that in some cases, cells can actually inhibit each other through electric field effects (Blot and Barbour, 2014); this tendency may be related to the anti-synchronous locking we observe here. More generally, this example demonstrates the relative simplicity by which a simulation can be conceived, implemented and analyzed using E_L_FENN while avoiding the technical challenges involved in implementing a cable equation solver along with electric field effects.

## Discussion

Here, we have presented E_L_FENN, a MATLAB toolbox for modeling ephaptic coupling. We have modeled ephaptic coupling in a standard way: by modeling the membrane potential and LFP and then closing the loop by feeding the extracellular potential back to the membrane potential dynamics through an ephaptic current (Holt and Koch, 1999). We have validated each component of our implementation against NEURON (Carnevale and Hines, 2006) and LFPy (Lindén et al., 2014). Finally, we have presented two simple use cases from the literature (Ramón and Moore, 1978; Jefferys, 1995; Goldwyn and Rinzel, 2016) to illustrate how ephaptic coupling can be modeled in realistic neuron models and how it can lead to phase-locked firing between adjacent axons.

There is of course room for improvement. Currently E_L_FENN is restricted to an isotropic (conductivity is as scalar) and homogeneous (at every point in space the conductivity is the same) extracellular space. There already exist formalisms for removing these simplifications (Nicholson and Freeman, 1975; Ness et al., 2015), which are planned for future development. Already without these features, E_L_FENN can be used to study a wide range of problems involving ephaptic coupling and explore the conditions in which electric field effects can influence on-going brain dynamics. In addition, E_L_FENN can also be used to model extracellular stimulation (where the electrode is another current source), as long as electrodes obey simple geometries.

Furthermore, the primary limitation of E_L_FENN is being locked to a single processor in MATLAB. Moving to compiled code with standard numerical libraries (e.g., SUNDIALs) may have a large developmental cost but would have large payouts as code parallelization would be possible and thus high-performance computing resources could greatly reduce the present limitations of E_L_FENN.

## Data Availability

E_L_FENN is available on Github at: https://github.com/ELFENN/ELFENN.

## Author Contributions

AS and JL conceived and designed the E_L_FENN platform. AS wrote E_L_FENN software with input from JL. AS and JL wrote the manuscript. All authors read and approved the final manuscript.

### Conflict of Interest Statement

The authors declare that the research was conducted in the absence of any commercial or financial relationships that could be construed as a potential conflict of interest.
